# Switching perception of musical meters by listening to different acoustic cues of biphasic sound stimulus

**DOI:** 10.1371/journal.pone.0256712

**Published:** 2021-08-30

**Authors:** Sotaro Kondoh, Kazuo Okanoya, Ryosuke O. Tachibana

**Affiliations:** 1 Department of Life Sciences, Graduate School of Arts and Sciences, The University of Tokyo, Tokyo, Japan; 2 Center for Evolutionary Cognitive Sciences, Graduate School of Arts and Sciences, The University of Tokyo, Tokyo, Japan; 3 RIKEN Center for Brain Science, Saitama, Japan; Medical University Hannover; Cluster of Excellence Hearing4all, GERMANY

## Abstract

Meter is one of the core features of music perception. It is the cognitive grouping of regular sound sequences, typically for every 2, 3, or 4 beats. Previous studies have suggested that one can not only passively perceive the meter from acoustic cues such as loudness, pitch, and duration of sound elements, but also actively perceive it by paying attention to isochronous sound events without any acoustic cues. Studying the interaction of top-down and bottom-up processing in meter perception leads to understanding the cognitive system’s ability to perceive the entire structure of music. The present study aimed to demonstrate that meter perception requires the top-down process (which maintains and switches attention between cues) as well as the bottom-up process for discriminating acoustic cues. We created a “biphasic” sound stimulus, which consists of successive tone sequences designed to provide cues for both the triple and quadruple meters in different sound attributes, frequency, and duration. Participants were asked to focus on either frequency or duration of the stimulus, and to answer how they perceived meters on a five-point scale (ranged from “strongly triple” to “strongly quadruple”). As a result, we found that participants perceived different meters by switching their attention to specific cues. This result adds evidence to the idea that meter perception involves the interaction between top-down and bottom-up processes.

## Introduction

Meter is one of the core features of music. Meter perception is a hierarchical grouping of beats, which regularly assigns subjective intensity to beats [1–6]. The most common meters include the duple, triple, and quadruple meters. The duple meter (heard in marching music, for example) has two beats, such as “s-w-s-w…” (s: strong/w: weak), while the triple meter (e.g., waltzes) has three beats “s-w-w-s-w-w-…”. The quadruple meter consists of four beats with medium-strong (ms) beats “s-w-ms-w-s-w-ms-w…”. One can recognize these emphatic (strong) beats, and thus sense the meter even if the sounds do not exhibit clear differences among them in actual acoustics.

Previous studies have suggested that meter perception consists of not only bottom-up processes on acoustical cues but also of top-down attentional processes. We perceive beat strength (also called “accent”) from acoustic features of the sound event (such as pitch, duration, and loudness) and recognize the strongest beat as the first of its meter cycle [7–10]; i.e., higher, longer, or louder sound events tend to be perceived as the accented beats. Furthermore, we can perceive meter by paying attention to isochronous beats without any physical accent [11–16]. Attending to rhythms without any body movements can modulate the motor area of the brain [17–19], which suggests that the top-down process involves rhythm production.

Musical expertise appears to play an important role in the active processes of meter perception. Even non-musicians can perceive meter, but musicians can perceive it more accurately and stably [11,20–23]. This active top-down process in meter perception has been conceptualized as the dynamic attending theory of meter perception [24–26], which places great importance on the ability to predict the timing of future events based on past sound events. This ability is especially important for extracting beat regularity, which requires an interaction between top-down and bottom-up processes. Hence, this theory posits that the perceptual strength of the beats reflects the allocation of attention.

Typical examples of the interaction between top-down and bottom-up processing can be seen in figure-ground reversals within the auditory stream segregation of sound sequences. Listeners can perceive sound series as multiple streams when their duration and frequencies are in a certain range. The number of streams can be switched according to the interplay of attention and acoustic features [27–30]. These studies on the interaction between top-down and bottom-up processes motivated us to ask whether meter perception might also switch depending on the attribute of sound events that one focuses on. Studying this interaction leads to understanding the cognitive system’s capacity for perceiving the structure of music.

The present study aimed to assess the effect that switching one’s attentive focus between different acoustic features has on the perception of different meters, as well as its extent. To this end, we used a novel sound stimulus that consisted of successive sound sequences. We named it the ‘biphasic stimulus.’ By design, its purpose is to produce the perception of either a triple or a quadruple meter depending upon which acoustical cues the listeners fixed their attention, whether it was frequency or duration. We asked participants which of two meters they identified more strongly in the stimulus, in order to test whether they could perceive different meters by switching the sound attributes they focused on. Additionally, we preliminarily assessed how the switch of meter perception was modulated by stimulus acoustical parameters and participants’ experience to be exposed to the biphasic stimuli beforehand.

## Methods

### Participants

A total of 16 participants (6 males; 24.5 ± 3.8 years old) participated in the study. None of the participants were professional musicians, though some participants had received musical practices as amateurs (10 participants, 7.4 ± 5.2 years). No participants reported a history of hearing difficulty. All participants have signed written informed consent. The consent form and all other experimental procedures were approved by the Ethics Review Committee on Experimental Research with Human Subjects of the Graduate School of Arts and Sciences, The University of Tokyo, Japan (#580).

Note that a half of the participants had experienced the biphasic stimuli before the present study. We classified participants into three groups to assess the effect of the prior experiences, while we did not perform the statistical test on this ad hoc assessment. Eight participants listened to the stimuli for the first time in this study (labeled as ‘unexperienced’), but the other eight experienced the stimuli in the pilot experiment (‘experienced’; two participants) or in the training where they tap along to the sound (‘trained’; six participants) before starting the current experiment (Table 1). Details of the pilot experiment and the training were described in later section.

**Table 1:** Summary of participants’ information.

group	*n*	# male: female	age (years old)	# amateur musicians	duration of practice as amateur (years)
unexperienced	8	3:5	24.6 ± 5.3	4	9.8 ± 5.8
experienced	2	1:1	25.5 ± 2.1	2	6.0 ± 5.7
trained	6	2:4	24.0 ± 1.3	4	5.8 ± 5.0

### Stimuli

**Fig 1:**
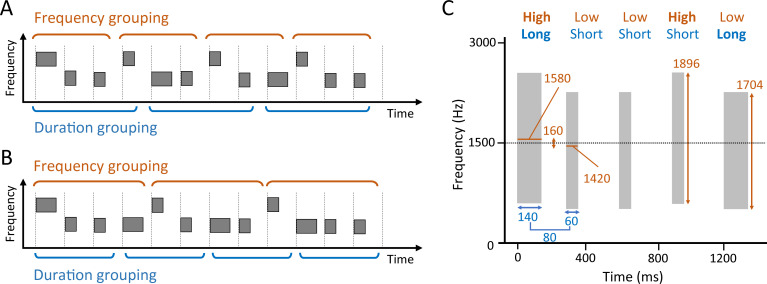
Schematic drawings of the biphasic meter stimuli. The stimuli consisted of various types of band-limited noise bursts. Inter-onset intervals were fixed at 300 ms. **A.** One type of stimulus had triple-meter regularity in the center frequencies of the noise bursts (high-low-low) and a quadruple cycle in their temporal durations (long-short-short-short). **B.** Another type of stimulus had the opposite property; triple and quadruple meters were assigned to durations (long-short-short) and frequencies (high-low-low-low), respectively. **C.** Schematic spectrogram of the former type of stimulus (shown in panel A) when the frequency difference is 160 Hz and the duration difference is 80 ms (F160-D80 condition). The bandwidth was 120% of the center frequency.

We designed two types of stimuli with sound sequences consisting of various types of band-limited noise bursts, and were designed to contain acoustical cues for two different meters (triple and quadruple) at the same time (Fig 1). One type of stimulus had a triple-meter cycle in center frequencies of the noise bursts (high-low-low) and a quadruple cycle in their temporal durations (long-short-short-short) (Fig 1A). Another type of stimulus had the opposite property, in which triple and quadruple meters were assigned to durations and frequencies, respectively (Fig 1B). In other words, we designed triple-frequency quadruple-duration stimulus and triple-duration quadruple-frequency stimulus. The inter-onset interval (IOI) of noise bursts was fixed at 300 ms.

We created five conditions by manipulating the difference in center frequency of the high and low noises from the baseline frequency of 1500 Hz. These differences were 102, 128, 160, 200, and 250 Hz, respectively. Thus, the center frequency of high noise was 1580 Hz when the frequency difference condition was 160 Hz (= 1500 + 160/2), while that of low noise was 1420 Hz (= 1500–160/2). We named these five frequency conditions by combining “F” with the digits for frequency difference (e.g., “F102” for the condition with 102 Hz of frequency difference).

We also set five conditions by manipulating the difference in duration between long and short bursts of noise from the baseline duration of 100 ms. The differences were 51, 64, 80, 100, and 125 ms. Therefore, when the difference was 80 ms, the duration of the long burst was 140 (100 + 80/2) ms, and that of the short burst was 60 (100–80/2) ms. Similar to frequency conditions, the duration condition was labeled as a combination of “D” and duration differences in milliseconds (consequently, “D51” refers to a 51-ms difference condition).

Frequency and duration differences in our stimuli were in the range that participants explicitly perceive them when these differences were presented respectively in the frequency or duration domain. The discrimination threshold of the center frequency of the band noise is known to be approximately 3–16 Hz when the center frequency is 2000 Hz [31], while the minimum frequency difference in the present study was 102 Hz. On the duration difference, the discrimination threshold has been reported as around 10 ms for a 100 ms noise-burst [32], which is much shorter than our minimum duration difference of 51 ms.

The bandwidth of each noise burst was 120% of the center frequency, and the rise/decay time was 10 ms. In total, we prepared 25 different stimuli (5 frequency × 5 duration conditions) for each of the two types of the meter assignment (frequency-triple vs. duration-triple type). Each stimulus consisted of 12 noise bursts, resulting in a total duration of 3.6 s.

### Procedure

The task of participants was to evaluate how well they identified the quadruple meter in a stimulus by pressing buttons corresponding to a five-point scale; 1: “strong triple”, 2: “triple rather than quadruple”, 3: “neither triple nor quadruple”, 4: “quadruple rather than triple”, and 5: “strong quadruple”. In one trial, we instructed a participant to focus on either frequency or duration by showing the phrase “height difference” or “duration difference” on a PC monitor for 0.8 s, and then we presented one stimulus twice successively. After collecting the response from the participant, we presented the next trial immediately. One whole trial was around 10 s in duration. We presented 100 trials (25 conditions × 2 types × 2 instructions) in random order as one block, and we conducted six blocks per participant. There was a break between blocks lasting at least five minutes.

The experiment was programmed and run with the use of Presentation 18.1 (Neurobehavioral Systems, Inc., USA). Visual stimuli were presented on a 21-inch LCD monitor (EIZO FlexScan L797; Eizo, Japan). The distance between the participant and the monitor was 80 cm. Auditory stimuli were generated using MATLAB R2017a (MathWorks) and presented binaurally at approximately 70 dB SPL via external soundcard (UA-22, Roland, Japan) and earphones (Earphone Insert, Compumedics Neuroscan Inc., USA). We instructed the participants to perform the task without any synchronized body movements such as finger tapping or head nodding. Immediately before starting the experiment, unexperienced participants listened to stimuli of the F250 condition with no duration difference as well as of the D125 condition without any frequency differences in order to learn to identify differences of frequency and duration in stimuli.

### Participants’ prior experience

The experienced group (two participants) joined the pilot experiment before the present study. We presented them with the biphasic stimuli: the frequency condition consisted of F100, F125, F158, F200, and the duration condition included D50, D63, D79, D100. IOI for each noise burst was 300 ms, and the bandwidth was fixed as 2000 Hz. The procedure was the same as the current study. We instructed which feature should be focused on (height or duration difference), then presented one stimulus. Participants rated how well they perceived the quadruple meter in the presented stimulus using a scale of 1 (strong triple) to 5 (strong quadruple). The participants rated the frequency-triple stimulus on day 1 and the duration-triple on day 2.

The trained group (six participants) practiced focusing attention on one acoustic cue (frequency or duration) by listening to the stimulus and accompanying it with hand tapping. At first, we presented them with a simple stimulus, which only differed in one feature. Then, we presented stimuli that included an additional difference. Participants understood the difference in frequency through practice by listening and tapping along to the F250-D0 condition first. Then, we presented them with stimuli of the F250-D51, F200-D64, and F160-D80 conditions, in that order. They tapped along to the sound while paying attention to the frequency difference; this helped them understand the meter [14,16,33,34]. If they reported that they could fully pay attention to the frequency difference of one stimulus, we presented the next stimulus. In the training of attention to the duration difference, we presented the participants with F0-D125, F102-D125, F125-D100, and F160-D80 conditions, in that order. The training took between 30 minutes to an hour, depending on the participant.

### Analysis

To assess whether the participants perceived different meters by switching their attention between acoustic features, we categorized data as “triple-targeted” or “quadruple-targeted” trials in which participants paid attention to the acoustic feature with the regularity of triple or quadruple meter, respectively. We calculated the difference of subjective rating scores between the triple- or quadruple-targeted trials for each acoustical condition. This difference should be larger when the participant successfully perceived different meters by switching their attention between two acoustic features. For assessing the effect of attentional switching, we averaged the rating difference data across all trials by participant. To test whether the rating difference was significantly different from zero, we performed a one-sample Wilcoxon signed-rank test with the significance level *α* at 0.05.

We supplementarily assessed how much the frequency and duration conditions modulated the switch of the perceived meter. For this aim, we used data obtained only from participants who exhibited clear switches in the meter rating (11 participants). We quantified slopes of the frequency and duration factors by fitting a linear mixed-effects regression (LMER) model. The rating difference was set as a response variable, and frequency, duration, and the interaction between them were included as fixed effects in the model. Note that we centered the frequency and duration values by subtracting their means. The participant index was used as a random effect. Thus, the model expression we used here was as follows: *rating_difference ~ frequency + duration + frequency×duration +* (*1|subject*). We performed the analysis in R version 4.0.4 [35]. To fit the model, we used ‘lmer’ function of the lme4 package [36]. We also adopted ‘r.squaredGLMM’ function of the MuMin package [37] to calculate the LMER’s fitted ness with *R*-squared value [38].

To analyze the effect of participants’ prior experience, we only reported the individual rating difference because we have not designed our experiment to test the statistical significance of the experience effect.

## Results

Previous studies have suggested that meter perception involves the interaction between top-down and bottom-up processes. According to this idea, we expected that the perceived meter could switch depending on the regularity of the stimulus attribute that the listener focused on. Thus, the present study aimed to examine whether a listener could perceive meter by switching their attention across different acoustic features and, if so, to what extent.

**Fig 2:**
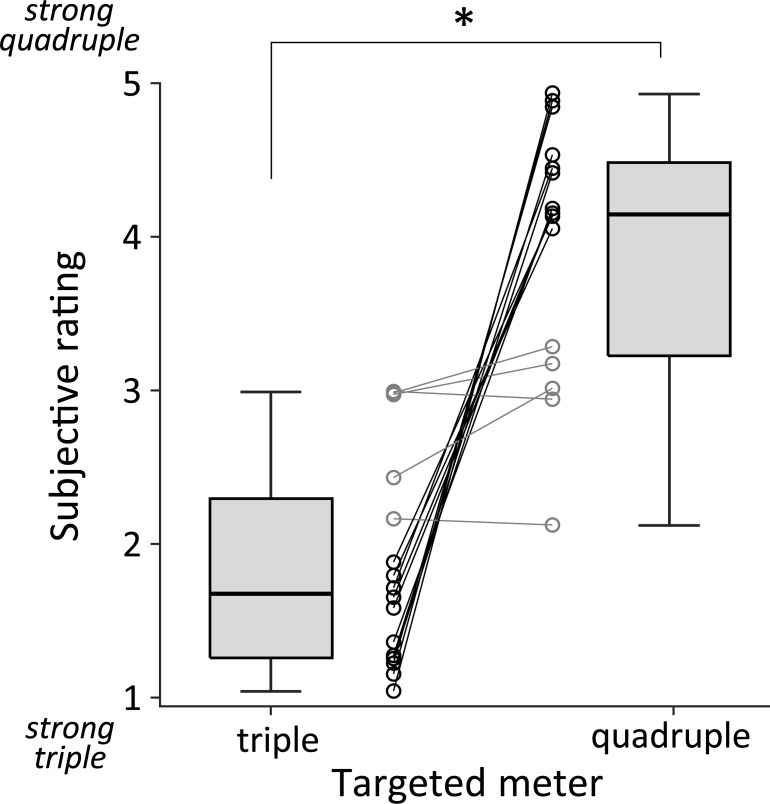
Mean subjective rating of triple- and quadruple-targeted trials. Participants (*n* = 16) rated perceived meters from the biphasic stimuli on a five-point scale (from 1: “strong triple” to 5: “strong quadruple”). The rating score was relatively lower (more triple) for triple-targeted trials, and higher (more quadruple) for quadruple-targeted ones, suggesting that the listeners perceived different meters depending on the acoustic features which they attended. Gray circle and line indicate the five non-switchers. Asterisk (*) shows the significant difference (*p* < 0.05).

We designed the biphasic sound stimuli to contain two different meters (triple and quadruple) for different acoustic features. The participants evaluated the subjective strength of the quadruple meter. We found that the rating scores were low in triple-targeted trials (mean ± SD; 1.84 ± 0.68) and high in quadruple-targeted trials (3.95 ± 0.81) (Fig 2). The one-sample Wilcoxon signed-rank test showed that the rating difference between quadruple- and triple-target trials was significantly different from zero (*n* = 16, *V* = 133, *p* < 0.001). Five out of 16 participants did not exhibit clear switching in the rating score (shown as gray circles in Fig 2). Removing these non-switchers data points emphasized the rating difference between two targeted trials (triple-targeted: 1.45 ± 0.29; quadruple-targeted: 4.43 ± 0.33). We confirmed that the rating difference after removing non-switchers was significantly different from zero (*n* = 11; *V* = 66, *p* < 0.001). These results suggest that the perceived meter changed due to a switch in the attentional focus on different acoustical features.

**Fig 3:**
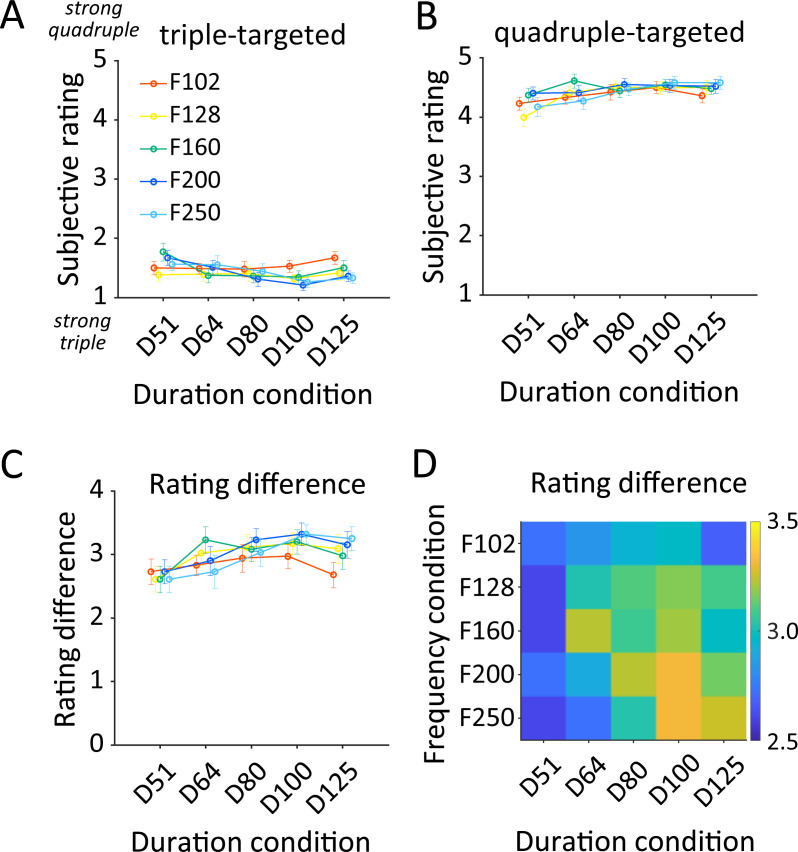
Subjective rating and rating difference for 25 conditions (frequency × duration conditions) in successfully switched participants (*n* = 11). **A,B.** Subjective rating of each condition focused on the acoustic feature which had (A) triple-meter or (B) quadruple-meter regularity. **C,D.** Rating difference between quadruple-targeted and triple-targeted trials as (C) line plots and (D) a color map. Error bar indicates the standard error across participants.

**Fig 4:**
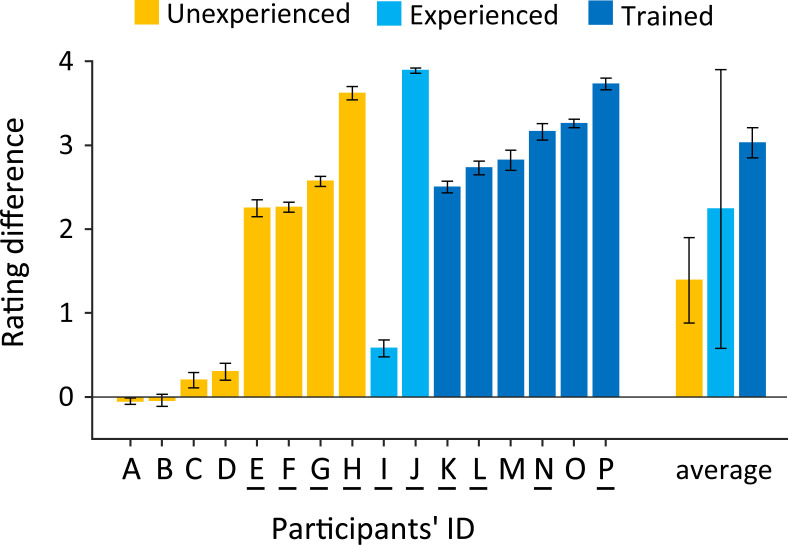
Rating difference and prior experience of each participant. The rating difference was averaged across all trials for each participant. Participants were classified into three group according to their prior experiences for listening to the biphasic stimuli (unexperienced, experienced, and trained; see details in Methods). Participants who had received musical practices are indicated by bars below IDs. Error bar of each participant indicates the standard error across 25 conditions (frequency × duration), and that of the group average shows the standard error across participants in that group.

We also explored how acoustical parameters of the biphasic stimuli effectively elicit different meter perceptions in the successfully switched participants (*n* = 11). The subjective ratings ranged from 1 to 2 in triple-targeted trials (Fig 3A) and 4 to 5 in quadruple-targeted trials (Fig 3B). The rating difference was slightly elevated when the duration difference increased irrespective of the frequency condition (Fig 3C and 3D). The largest difference was 3.32, obtained when the frequency and duration conditions were F200-D100 and F250-D100. On the other hand, the smallest difference was 2.61 acquired in the following conditions: F128-D51, F160-D51, and F250-D51. The LMER model fitting showed linear increases of the rating difference as functions of frequency and duration with the slopes of 0.89×10^−3^ points/Hz and 4.82×10^−3^ points/ms, respectively (Table 2). The interaction term had a slope of 0.06×10^−3^. These results indicate that a change in the frequency condition from F102 to F250 (148 Hz increase) raises the rating difference by 0.13 points, and a prolongation in the duration condition from D51 to D125 (74 ms) elevates the rating difference by 0.36 points. The model fitting performance was moderate (*R*^2^ = 0.74). Such small slopes were caused partially due to nonlinearity in the pattern of rating difference scores, in which the score rose with the duration increase but fell from D100 to D125 (Fig 3C), for example.

**Table 2:** Summary of the LMER model fitted to the rating difference for 25 conditions in switchable participants (*n* = 11).

Variable	*slope*	*SE*	*df*	*t*-value
frequency	0.89 × 10^−3^	0.41 × 10^−3^	261	2.18
duration	4.82 × 10^−3^	0.81 × 10^−3^	261	5.91
frequency × duration	0.06 × 10^−3^	0.02 × 10^−3^	261	3.62

We had noticed a large variability in the rating score across participants in pilot studies. This fact appeared to be explained that the perceptual switch depends on the prior experience with the stimuli and that more intentional training enables one to switch successfully. As an additional exploration in the present study, we trained a subset of the participants (*n* = 6) before starting the current experiment. Note that we did not have a clear hypothesis on the prior experience effect at this point, and hence, this exploration was not statistically well-designed. Thus, we here described the result just as a case report. We classified them into three groups (unexperienced, experienced, trained) and calculated the individual rating differences (Fig 4). Results demonstrate that all trained participants were able to switch perceive meters successfully (6 out of 6), while only the half of the unexperienced and experienced participants succeeded (unexperienced: 4 out of 8, experienced: 1 out of 2). Although the prior experience for listening to the stimuli (without the training) seems not to contribute to the extent of switching, all trained participants switched and perceived different meter. This result is consistent with an idea that enhanced top-down attention on beats facilitates the meter perception.

## Discussion

The present study investigated whether it was possible to perceive different meters by switching one’s attention to different acoustic features in the sound stimulus designed to contain triple or quadruple periodicity in frequency and temporal duration. We found that participants perceived meters differently depending on the sound attribute what they paid attention to (Fig 2). The result suggests that meter is perceived not only through bottom-up processes (by differentiating acoustic cues) but also through interactions with top-down processes (by paying attention to them).

We supplementarily explored how changes in the acoustic parameters influence metric perception in the biphasic stimulus. The analysis allows us to know mainly two things. First, the LMER shows the quantitative descriptions of each effect’s slope (Table 2), which indicates the extent of increasing attentional switch between two meters along with a unit increase of the frequency or duration difference of the biphasic stimulus. Second, the pattern of the rating difference (Fig 3C and 3D) implies what the optimal condition would be. As results, the rating difference was not entirely linear with increasing frequency and duration conditions: the difference was higher for the F200-D100 and F250-D100 conditions than for the F250-D125, raising the possibility that not merely increasing both frequency and duration does not facilitate meter perception in the stimuli and that there are the appropriate parameters. These effects of manipulating the acoustical features should depend on the range of frequency and/or duration that we arbitrarily chose after a pilot study. Note that the differences in frequency and duration were much greater than differential thresholds reported in previous studies (see Stimuli in Methods) [31,32]. Future studies should address the effects of extended ranges of acoustic features.

As preliminary results, we found that half of the participants in the unexperienced and experienced groups showed switching perception, whereas all participants in the trained group successfully switched (Fig 4). These findings suggest that training with sound-synchronized tapping, rather than passive listening to the stimuli, enhances top-down processes for meter perception, perhaps by explicitly directing listeners’ attention to the appropriate cues. Tapping to beats enhances detecting whether the probe tone after a short silence coincided with the timing of preceding beats [33,34], and listening to identical beats with tapping on every two or three of them facilitates duple or triple meter perception [14,16]. These previous reports suggest that body movements synchronized with regular-interval sound sequences enhance the ability to perceive the metric structure in the sound sequence. Interestingly, four unexperienced participants who had received musical practice (see Fig 4, participants E, F, G, and H) showed greater contrasts in the rating scores across triple- and quadruple-targeted trials comparing to the others in the unexperienced group without practice (participants A, B, C, and D). Since musical expertise has reported improving meter perception accuracy [11,20–22], participants’ musical practices might also influence the individual differences in top-down processing in our experiment. The influence of prior experiences and the training on meter perception of the biphasic stimulus needs to be further assessed in future studies.

Further, the present study would provide an implication that meter perception is rooted in an active process of identifying subjective regularities in the inputting auditory stream. At least two-thirds of participants perceived the targeted meter by switching their attention to the acoustic features, suggesting that they can selectively recognize the specific regularity in ambiguous sound sequences. This ability might be related to more general auditory attention, e.g. the cocktail-party effect; selective listening to a specific acoustical regularity of speech sounds against background noises [39]. Such general auditory ability could share a common foundation with the meter perception of the biphasic stimulus, and this issue should be addressed in future researches.

This study employed triple and quadruple meters for the biphasic stimuli, instead of duple meter, which is one of the most typical meters. This is because we intended to avoid the risk of participants perceiving a sextuple meter (grouping as a set of six beats) from the stimulus containing triple and duple meters. In fact, we confirmed that several volunteers have reported hearing a sextuple meter in such stimuli during our pilot study. The biphasic stimulus for triple and duple meters exhibited the most accented sound (high and long) in a six-sound cycle as the common multiple of two meters, and tends to sound as a sextuple meter at a relatively faster tempo. Even for isochronous sound sequences (with no physical accent), participants hear the grouping of six sounds when the sound-interval is 300 ms or less [40,41]. In contrast, the combination of triple and quadruple meters (used in this study) potentially provides the cue for a twelve-beat meter, but it is rarer in music than the sextuple meter and has more elements that make up a group. Hence, it is difficult to perceive twelve-beat meter in our stimuli at the current tempo, IOI of 300 ms. We did not examine whether a change in tempo altered meter perception. According to a previous review [4], the range of IOI for meter perception is between 100 ms to 6 s. As discussed above, there could be a relationship between tempo and meter perception; the listener might perceive longer meters (more beats in one cycle) with a faster tempo, or shorter meters with a slower tempo. Future studies are required to address how the variety of meters from the biphasic stimuli with different tempo is perceived.

We developed the sound stimuli that has multiple acoustic features like actual music but is easier to control. Previous studies used short melodies and rhythmic sequences, which had similar patterns to real music pieces, in order for participants to perceive the meter [21,42]. These stimuli were not suitable for our purpose since they contained too many cues in different acoustical features, such as pitch, duration, and timbre. Our stimulus design (with a limited variety of acoustical cues) allowed us not only to assess the interaction directly but also to finely adjust the differences in frequency and duration in order to examine the changes in meter perception.

The present study demonstrated that participants controlled their attention to certain acoustic features for perceiving the meter. This provides an insight into how we perceive meter through the interaction between top-down-bottom-up processing. Furthermore, we contributed to assessing the key feature of musical expertise since our preliminary results suggest the possibility that the training leads to the enhancement of top-down processes for meter perception. Musicians produce and perceive the meter that guides their performance, and their top-down processing of meter perception should be stronger than that of non-musicians. Though further studies are necessary to sophisticate the training procedure for promoting meter perception, we believe that enhancements of the interaction between top-down and bottom-up processes must be the key for musical expertise, particularly in meter perception and production.

## Supporting information

S1 The F200-D100 condition of the biphasic stimulus which has quadruple-frequency triple-duration cycles. (WAV)

S2 The F200-D100 condition of the biphasic stimulus which has triple-frequency quadruple-duration cycles. (WAV)
